# Evaluation of CARBA PAcE, a novel rapid test for detection of carbapenemase-producing *Enterobacterales*

**DOI:** 10.1099/jmm.0.001290

**Published:** 2020-12-03

**Authors:** Janko Sattler, Anne Brunke, Axel Hamprecht

**Affiliations:** ^1^​ Institute for Medical Microbiology, Immunology and Hygiene, University of Cologne, University Hospital of Cologne, Cologne, Germany; ^2^​ DZIF (German Centre for Infection Research), partner site Bonn-Cologne, Cologne, Germany; ^3^​ Institute for Medical Microbiology and Virology, University of Oldenburg, Oldenburg, Germany

**Keywords:** CARBA PAcE, carbapenem, carbapenemase-producing *Enterobacterales*, carbapenemase, colorimetric test, multiresistant *Enterobacterales*

## Abstract

**Introduction:**

Carbapenemase-producing *Enterobacterales* (CPE) are an increasing threat to global health. Fast detection is crucial for patient management and outbreak control.

**Hypothesis/Gap statement:**

Recently, a new commercial colorimetric test, CARBA PAcE, was released that has not yet been scientifically evaluated.

**Aim:**

Our goals were to evaluate the performance of CARBA PAcE using a large variety of different CPE.

**Methodology:**

CARBA PAcE was challenged with 107 molecularly characterized CPE and 53 non-CPE controls. Isolates were grown on Mueller-Hinton agar (MHA); in the case of a false-negative result, isolates were additionally inoculated on Columbia blood agar (CBA) and CARBA PAcE was repeated. The test was performed according to the manufacturer’s protocol.

**Results:**

CARBA PAcE showed an overall sensitivity and specificity of 72 % [confidence interval (CI) 62–80 %] and 91 % (CI 79–97 %), respectively, when isolates were grown on MHA. With growth on CBA, detection improved (especially of metallo-β-lactamases), resulting in an extrapolated sensitivity of 89 % (CI 81–94 %) for all carbapenemases and 96 % (CI 89–99 %) for the four major carbapenemases (NDM, OXA-48-like, KPC, VIM).

**Conclusion:**

CARBA PAcE is a simple and very rapid test for the detection of CPE which performs well for the major carbapenemases when isolates are grown on CBA. Laboratories should be aware of the limitations of this assay, such as moderate sensitivity when isolates are grown on more challenging agars such as MHA and the poor detection of some rare carbapenemases (e.g. IMI, OXA-58).

## Introduction

Carbapenemase-producing *Enterobacterales* (CPE) are an increasing threat to global health [1]. Infections with CPE are associated with increased mortality [2] and nosocomial transmission due to vertical and horizontal gene transfer [3]. Therefore, further effort is needed to prevent further spread of CPE, and fast detection of CPE is indispensable for patient management and infection control [4].

Today, there is an increasing number of tests available for carbapenemase detection [5]. They can be classified into biochemical, phenotypic and molecular methods. The non-molecular methods include colorimetric tests, the carbapenem inactivation method and modifications thereof, immunochromatographic assays and matrix-assisted laser desorption ionization-time of flight MS-based tests. The assays differ widely in accuracy and turnaround time [6].

Colorimetric tests detect carbapenemase activity by hydrolysis of a β-lactam substrate, which subsequently leads to a pH shift that is visualized by a pH indicator. While early tests required manual preparation of the test reagents [7], today there are plenty of commercially available tests on the market that allow easy implementation in the diagnostic laboratory [8].

Recently, a new colorimetric test for rapid detection of CPE, CARBA PAcE (Mast Diagnostica), was released that has a turnaround time of only 10 min. It uses a novel chromogenic cephalosporin analogue along with inhibitors for extended spectrum β-lactamases (ESBLs) and AmpC β-lactamases. Details about the composition and biochemistry of the substrate have not been disclosed by the manufacturer.

This study aims to evaluate the performance of CARBA PAcE on a large number of CPE.

## Methods

CARBA PAcE was challenged with 107 molecularly characterized CPE and 53 non-CPE controls. The challenge collection comprised a broad spectrum of carbapenemase classes and subtypes including 27 isolates of NDM, 23 OXA-48-like, 19 KPC, 17 VIM, nine IMI, four IMP, two OXA-58, one GES and five isolates with two carbapenemases. Details of the challenge isolates are listed in Table S1 (available with the online version of this paper). All isolates have been characterized by phenotypic and immunochromatographic tests as well as molecular methods as previously described [5, 9].

**Fig. 1.**
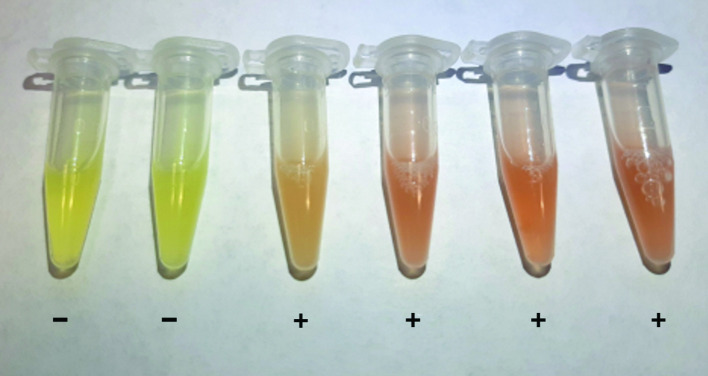
Example results of the CARBA PAcE test. The two samples on the left side were interpreted as negative (−), while the other samples shown were interpreted as positive (+) according to the manufacturer’s reading guide.

CARBA PAcE tests were performed according to the manufacturer’s recommendations. Briefly, bacterial isolates were cultured overnight at 37 °C on Mueller-Hinton agar (MHA) and, for isolates with false negative results, additionally on Columbia blood agar (CBA). A full 1 µl inoculation loop of bacterial colonies was transferred to the CARBA PAcE solution and dissolved by at least 20 s of vortexing until a homogeneous suspension was reached. In a pilot trial, we ensured that this inoculum meets the required turbidity equivalent to a McFarland standard of 3 or more (data not shown). After 10 min of incubation at 37 °C, the colour change of the solution was visually evaluated according to the manufacturer's reading guide. A colour change from yellow to orange or red was interpreted as positive (Fig. 1). The colour was evaluated again 10 min later. Reading was done in a blinded manner, i.e. the reader was unaware of the species and carbapenemase. When a result was unclear at the first read, the sample was re-cultivated overnight and the CARBA PAcE test was repeated. For the re-evaluation, a consensus was sought between two readers and this was determined as the final result.

Sensitivity, specificity and the 95 % confidence interval (CI) were calculated using molecular results as a reference. Isolates with agreement between molecular characterization and the CARBA PAcE result were tested once whereas the test was repeated for isolates with an ambiguous result. All isolates with false-negative results from MHA were retested after subculture on CBA. Because only false-negatives were tested after culture on CBA, the overall sensitivity of CARBA PAcE from CBA was calculated from the sum of true positives tested from CBA and MHA divided by the total number of CPE isolates. CPE harbouring more than one type of carbapenemase were included for the calculation of overall sensitivity, but not considered in the subgroup analysis.

## Results

CARBA PAcE correctly detected 77/107 (72 %, CI 62–80 %) CPE when isolates were grown on MHA, and 48/53 (91 %, CI 79–97 %) non-CPE tested as true negative. False negative samples were additionally tested after cultivation on CBA, which resulted in an improved overall sensitivity of 89 % (CI 81–94 %). Notably the sensitivity for Ambler class B and D carbapenemases improved from 67 % (CI 52–80 %) to 94 % (CI 83–99 %) and from 80 % (CI 59–93 %) to 92 % (CI 74–99 %), respectively, whereas sensitivity for class A carbapenemases hardly changed (72%, CI 53–87 % vs. 76 %, CI 56–90 %) (Table 1). The poor sensitivity for class A carbapenemases was attributable to IMI carbapenemases for which CARBA PAcE showed a sensitivity of only 11 % (CI 0–48 %) or 22 % (CI 3–60 %), depending on the culture medium. In contrast, KPC carbapenemases were well detected (sensitivity 100 %, CI 82–100 %). Results of CARBA PAcE tests were consistent when read directly or 10 min after the incubation time proposed by the manufacturer. Six isolates gave rise to an ambiguous result initially and had to be repeated for re-evaluation, resulting in four true positive and two false negative results.

**Table 1.** Test sensitivity of CARBA PAcE compared to molecular methods with isolates grown on Mueller-Hinton agar (MHA) or Columbia blood agar (CBA) according to carbapenemase type

Carbapenemase type	Sensitivity (MHA)	Sensitivity (CBA)
**Class A (*n*=29**)	**72 % (CI 53–87 %)**	**76 % (CI 56–90 %)**
GES (*n*=1)	100 % (CI 3–100 %)	100 % (CI 3–100 %)
IMI (*n*=9)	11 % (CI 0–48 %)	22 % (CI 3–60 %)
KPC (*n*=19)	100 % (CI 82–100 %)	100 % (CI 82–100 %)
**Class B (*n*=48)**	**67 % (CI 52–80 %)**	**94 % (CI 83–99 %)**
IMP (*n*=4)	100 % (CI 40–100 %)	100 % (CI 40–100 %)
NDM (*n*=27)	63 % (CI 42–81 %)	96 % (CI 81–100 %)
VIM (*n*=17)	65 % (CI 38–86 %)	88 % (CI 64–99 %)
**Class D (*n*=25)**	**80 % (CI 59–93 %)**	**92 % (CI 74–99 %)**
OXA-48 (*n*=6)	100 % (CI 54–100 %)	100 % (CI 54–100 %)
Other OXA-48-like (*n*=17)	76 % (CI 50–93 %)	94 % (CI 71–100 %)
OXA-58 (*n*=2)	50 % (CI 1–99 %)	50 % (CI 1–99 %)

## Discussion

This is, to the best of our knowledge, the first systematic evaluation of the CARBA PAcE test. The overall performance was moderate with a sensitivity and specificity of 72 and 91 %, respectively, with isolates grown on MHA. The performance was below average values for other colorimetric tests, with large comparative studies reporting sensitivity ranges of 89–98 % and specificity ranges of 84–100 % for the Rapidec Carba NP (bioMérieux), Neo-Rapid Carb kit and Rapid Carb Blue screen (both Rosco) [6, 10]. In contrast to the present study, however, these studies included only few or no IMI carbapenemase-producing isolates, which contribute strongly to the moderate performance of CARBA PAcE in this study.

The performance of the CARBA PAcE assay is comparable to the β-Carba test (Bio-Rad), with a sensitivity of 74 % [5]. As in the latter study, results could be significantly improved when false negative isolates were cultured on CBA instead of MHA. With the resulting sensitivity of 89 % (CI 81–94 %) in the present study, the CARBA PAcE test is at the lower average of the colorimetric test performance range. Poor detection rates for NDM and VIM (but not IMP) carbapenemases are a known problem when isolates are cultured on MHA and have been reported for other colorimetric tests [5, 11] and immunochromatographic assays [12]. The reason for the poor detection of MBL carbapenemases is an insufficient zinc concentration in these media, with zinc being an important co-factor for metallo-β-lactamase activity [13]. This can be overcome by either supplementing the agar with zinc or using blood agar with a naturally higher zinc concentration [5, 12].

Apart from the poor performance for NDM and VIM carbapenemases in isolates cultured on MHA, CARBA PAcE showed low detection rates for IMI carbapenemases. This is probably due to the lower hydrolysing activity of these enzymes [14]. Given their low prevalence, these carbapenemases are included in only a few evaluation studies, and the performance of other colorimetric tests was also poor [5, 15]. IMI carbapenemases are rarely detected in Europe and the USA. Nevertheless, this limitation must be kept in mind when this test is used in regions where IMI carbapenemases are frequent.

False positive results were noted in five non-CPE isolates including of *Escherichia coli* (*N*=4) and *Serratia marcescens* (*N*=1). All false positive strains were ESBL producers. False positive results due to ESBL production have been observed in other colorimetric tests [15]. Furthermore, *S. marcescens* is recognized as a problematic species for CARBA PAcE in the company’s FAQ sheet as it can produce coloured pigments which may hamper the specificity of the test.

CARBA PAcE has a very low turnaround time of only 10 min. Further incubation time did not improve the final result. Turnaround times for other colorimetric tests range from 30 min (β-Carba test) to 120 min (Rapidec Carba NP, final reading). Therefore, CARBA PAcE is to date the fastest colorimetric test available. Implementation in the routine laboratory is easy, as only standard laboratory equipment is required. A shortcoming that CARBA PAcE shares with other colorimetric tests is the subjective nature of test interpretation. Furthermore, carbapenemases cannot be further classified with this test, in comparison with immunochromatographic or molecular assays.

A limitation of this study is the choice of isolates, which do not represent the true epidemiological distribution of carbapenemases. As the tests were challenged with a broad variety of carbapenemases including rare and difficult to detect carbapenemase types, and these carbapenemases are overrepresented in the challenge collection. Therefore, performance in routine diagnostic tests, where the four most common carbapenemases (KPC, NDM, VIM, OXA-48-like) prevail, would be expected to be better. Another limitation is the choice of MHA as the initial culture medium, as detection of metallo-β-lactamases is more difficult. MHA was chosen as this probably best reflects the situation in the diagnostic laboratory, where carbapenem resistance is often detected in agar diffusion tests performed on MHA and subsequent confirmation tests are then performed from this medium. Furthermore, apart from chromogenic and selective media, no restriction of growth media was specified in the product manual. To overcome this limitation, we also performed the CARBA PAcE test on isolates subcultured on CBA for the false negative test results. When isolates are cultured on CBA and only the four most common carbapenemase types were included, the extrapolated sensitivity increased to 96 % (CI 89–99 %), which is similar to the performance values provided by the manufacturer. Future studies should further analyse the performance of CARBA PAcE for carbapenem-resistant *Pseudomonas* or *Acinetobacter* species, which can also be tested by this assay.

In conclusion, this is the first systematic evaluation of the CARBA PAcE test. The advantage of CARBA PAcE is the low turnaround time of 10 min, albeit at the cost of a moderate performance. To achieve good sensitivity, it should strictly be used for isolates grown on media with high zinc content.

## Supplementary Data

Supplementary material 1
