# Supplementary material for: Evaluation of CARBA PAcE, a novel rapid test for detection of carbapenemase-producing Enterobacterales
Source: J Med Microbiol. 2020 Dec 3;70(2):001290. doi: 10.1099/jmm.0.001290 (PMC8131015; doi:10.1099/jmm.0.001290)
Supplement: Supplementary material 1 [file jmm-70-290-s001.pdf]

Evaluation of CARBA PAcE, a novel rapid test for detection of carbapenemase producing Enterobacterales

Janko Sattler [a,b], Anne C. Brunke [a,b], Axel Hamprecht [a,b,c, #]

[a]Institute for Medical Microbiology, Immunology and Hygiene, University of Cologne, University Hospital of Cologne, Cologne, Germany

[b]DZIF (German Centre for Infection Research), partner site Bonn-Cologne, Cologne, Germany

[c]Institute for Medical Microbiology and Virology, University of Oldenburg, Oldenburg, Germany

[#]Address correspondence to Axel Hamprecht, axel.hamprecht@uol.de

Supplementary material

Supplementary table 1 Carbapenemase producing Enterobacterales (CPE) analyzed in this study, subclassified by species and carbapenemase subtype. The category others comprises *Klebsiella oxytoca* (VIM-4), *Raoultella ornithinolytica* (NDM-1) and *Morganella morganii* (control).

	K. pneumoniae	***E. coli***	***E. cloacae complex***	***C. freundii complex***	***K. aerogenes***	***S. marcescens***	***P. mirabilis***	***Others***
**All isolates**	**54**	**45**	**33**	**13**	**6**	**3**	**3**	**3**
**Carbapenemase positive**	**43**	**26**	**2**	**12**		**2**	**2**	**2**
**Class A**	**13**		**11**	**5**				
GES-25				1				
IMI-1			1					
IMI-2			1					
IMI-3			1					
IMI-4			1					
IMI-9			1					
IMI-10			1					
IMI-12			1					
IMI-14			1					
IMI-16			1					
KPC-2	9		2	1				
KPC-3	4			3				
**Class B**	**17**	**12**	**9**	**6**		**2**		**2**
IMP-1	1							
IMP-4	1							
IMP-8				1				
IMP-22	1							
NDM-1	8	4	3			1		1
NDM-3		1						
NDM-4		1						
NDM-5		2		1				
NDM-7		1	1					
NDM-8	1							

	K. pneumoniae	*E. coli*	*E. cloacae complex*	*C. freundii complex*	*K. aerogenes*	*S. marcescens*	*P. mirabilis*	*Others*
NDM-9	2							
VIM-1	1	3		1				
VIM-2	1			1				
VIM-4			2					1
VIM-26			1					
VIM-31				1				
VIM-39			1					
VIM-46	1							
VIM-54						1		
VIM-56				1				
VIM-58			1					
**Class D**	**10**	**12**		**1**			**2**	
OXA-48	2	4						
OXA-162	2	1		1				
OXA-181		4						
OXA-204	1							
OXA-232	1	2						
OXA-244	1	1						
OXA-245	2							
OXA-370	1							
OXA-58							2	
**CPE with 2 carbapenemases**	**3**	**2**						
KPC-2 + VIM-1	2							
NDM-1 + OXA-232	1							
NDM-5 + OXA-181		1						
VIM-1 + OXA-48		1						
**Negative controls**	**11**	**19**	**13**	**1**	**6**	**1**	**1**	**1**
